# Orthostatic Dysregulation during Postural Change on the Dental Chair and Intraoperative Monitoring by Heart Rate Variability Analysis

**DOI:** 10.1155/2014/656045

**Published:** 2014-06-17

**Authors:** Yukihiro Momota, Shigemasa Tomioka, Mayuko Furukita, Kenji Fujisawa, Hideyuki Takano, Masayuki Azuma

**Affiliations:** ^1^Department of Oral Medicine, Institute of Health Biosciences, The University of Tokushima Graduate Faculty of Dentistry, Kuramoto 3-18-15, Tokushima 770-8504, Japan; ^2^Department of Dental Anesthesiology, Institute of Health Biosciences, The University of Tokushima Graduate Faculty of Dentistry, Tokushima 770-8504, Japan; ^3^Department of Oral Surgery, Institute of Health Biosciences, The University of Tokushima Graduate Faculty of Dentistry, Tokushima 770-8504, Japan

## Abstract

This is the first case report of orthostatic dysregulation (OD) manifested during postural change on the dental chair and intraoperatively monitored by heart rate variability (HRV) analysis. OD-associated autonomic dysfunction is induced by postural changes and easily leads to disturbance in circulatory dynamics; however, most dental practices have not yet realized the importance of managing OD. We measured autonomic activity in a patient with OD during dental therapy and assessed the clinical significance of HRV analysis for OD. The patient was a 17-year-old Japanese female. She was diagnosed with impacted wisdom teeth and had no previous history of a distinct systemic disease. A surgical procedure to extract the teeth was safely performed under both local anesthesia and sedation with nitrous oxide and midazolam. After the surgery, her postural change to sitting induced orthostatic hypotension. HRV variables showed parasympathetic dominance due to the upright position. Subsequently, her posture was returned to supine, and atropine sulfate administration for the immediate treatment of OD returned her blood pressure to normal levels. HRV variables showed relative sympathetic dominance due to an atropine-derived parasympathetic blockade. HRV analysis revealed OD-associated autonomic dysfunction and should become a standard tool for safe and secure dental management of OD.

## 1. Introduction

Orthostatic dysregulation (OD) has been unjustly disregarded in spite of its serious symptoms: orthostatic intolerance, palpitation, syncope, dizziness, headache, abdominal pain, malaise, and so on [1–7]. Recently, OD has emerged as a social problem in the fact that adolescents with OD frequently refuse to attend school [8]. Unfortunately, previous medical approaches have not sufficiently addressed the problem. Furthermore, the etiology of OD is still unclear, even though OD-associated symptoms are considered a result of autonomic dysfunction [6, 9, 10]. Although criteria for diagnosing OD have been established, the criteria are not based on objective analysis but largely depend on symptomatology [11]. Heart rate variability (HRV) analysis is useful to assess autonomic activity and to diagnose autonomic neuropathy because HRV is biomarkers for the functions of the autonomic nervous system (ANS) [12–17]. In fact, abnormalities in daily HRV changes have been shown to be correlated with OD by HRV analysis [9]. An HRV analyzer looks just like a pulse oximeter and analyzes pulse-to-pulse variations in pulse rate by a built-in HRV analyzing system; it enables easy measurement of autonomic activity without inducing any stress in patients.

OD is generally regarded as an uncommon disease in dentistry. Postural changes which are likely to induce OD-associated autonomic dysfunction are performed during dental therapy. OD-associated autonomic dysfunction easily leads to disturbance in circulatory dynamics; however, most dental practices have not realized the importance of managing OD. Autonomic activity in OD during dental therapy has not previously been measured or elucidated. We experienced a rare case of OD manifested during postural change on the dental chair and intraoperatively monitored by HRV analysis; we assessed the clinical significance of HRV analysis for OD in a dental practice.

## 2. Case Report

**Figure 1:**
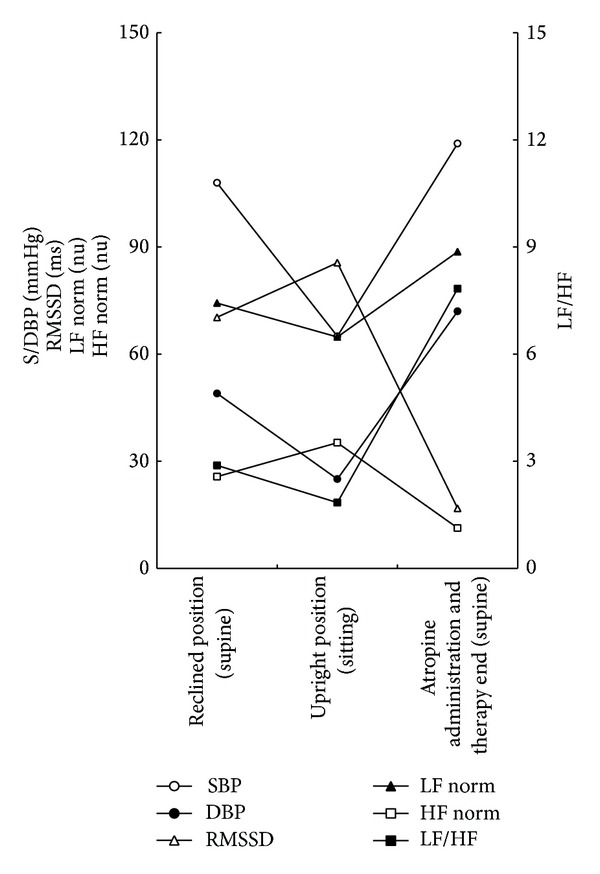
Time course of S/DBP and frequency-/time-domain HRV variables with postural change.

The patient was a 17-year-old Japanese female. She attended the Department of Oral Surgery and Dental Anesthesiology, Tokushima University Hospital. She was given the diagnosis of impacted wisdom teeth and had no previous history of a distinct systemic disease such as cardiovascular, cerebrovascular, or psychiatric disease, even though sinus bradycardia was pointed out on the preoperative electrocardiographic examination. However, she had previously developed surgery-phobia and experienced a vasovagal reflex while her blood was drawn. In advance of surgery, an intravenous line was established on her ulnar side with inhalation of 30.0% nitrous oxide and followed by local anesthesia, namely, infiltration of 3.6 mL of lidocaine containing 0.08% adrenaline accompanied by intravenous administration of 2.5 mg of midazolam. The surgical procedure to extract the teeth was safely performed. When her posture was changed from supine to sitting 40 minutes after the surgery, orthostatic hypotension (systolic/diastolic blood pressure (S/DBP): 65/25 mmHg) with nausea developed 5 minutes after the postural change (Table 1, Figure 1). Compensatory tachycardia and syncope were not found at the same time. Her posture was returned to supine; her blood pressure (BP) gradually increased to 106/55 mmHg. When her posture was changed to sitting 30 minutes later, hypotension (S/DBP: 66/33 mmHg) recurred 7 minutes after the postural change. After changing her posture back to supine, 0.5 mg of atropine sulfate was intravenously administered for the immediate treatment of OD, after which her BP returned to normal levels. Thereafter, her posture was changed to sitting; the hypotension did not recur, even though slight nausea was caused by decannulation.

HRV analysis was performed with an HRV analyzer (SA-3000P, Tokyo Iken Co., Ltd., Tokyo, Japan) during therapy. Concerning time-domain HRV variables, standard deviation of all NN intervals (SDNN) increased up to 193.0 ms; in contrast, approximate entropy (ApEn) decreased to 0.273 at the moment of local anesthesia (Table 1). When her posture was changed from supine to sitting, her mean heart rate (Mean HRT) and physical stress index (PSI) decreased; in contrast, root mean square of successive NN interval differences (RMSSD) increased. After changing her posture back to supine, atropine sulfate administration caused her Mean HRT and PSI to increase, in particular, for her PSI to increase up to 27.1 (Table 1). In contrast, RMSSD and ApEn decreased; in particular, RMSSD decreased to 16.8 ms (Table 1, Figure 1). Regarding frequency-domain HRV variables, at the onset of nitrous oxide inhalation, the high-frequency (HF) component increased to 1350.5 ms^2^; in contrast, normalized LF (LF norm) and the low-frequency/high-frequency (LF/HF) ratio decreased to 15.0 nu and 0.177, respectively (Table 1, Figure 1). The very low-frequency (VLF) component increased up to 4078.6 ms^2^ at the moment of midazolam administration (Table 1). At the moment of local anesthesia, the total power (TP), VLF, low-frequency (LF) component, LF norm, and LF/HF ratio increased up to 33920.5 ms^2^, 31187.1 ms^2^, 1919.1 ms^2^, 70.2 nu, and 2.357, respectively; in contrast, the HF norm decreased to 29.8 nu (Table 1). During teeth extraction, the HF increased up to 1906.0 ms^2^ (Table 1). At the end of nitrous oxide inhalation, the LF norm and LF/HF ratio increased up to 57.0 nu and 1.324, respectively (Table 1). In the recovery period, VLF decreased to 234.7 ms^2^; in contrast, LF/HF ratio increased up to 2.884 (Table 1). When her posture was changed from supine to sitting, TP, VLF, and HF increased up to 5215.6 ms^2^, 1695.0 ms^2^, and 1239.1 ms^2^, respectively (Table 1). Meanwhile, the LF norm relatively decreased, and HF norm increased such that the LF/HF ratio decreased. After changing her posture back to supine, atropine sulfate administration caused TP, LF, and HF to decrease to 1620.2 ms^2^, 426.9 ms^2^, and 54.5 ms^2^, respectively (Table 1). The LF norm relatively increased while HF norm decreased to 11.3 nu such that the LF/HF ratio increased to 7.833 (Table 1, Figure 1). At the end of therapy, all parameters returned to normal levels.

In advance of this study, the procedure for HRV analysis was explained to the patient. Then, her informed consent was obtained.

## 3. Discussion

To our knowledge, this is the first case report of OD manifested during postural change on the dental chair and intraoperatively monitored by HRV analysis.

Parameters calculated from HRV analysis are composed of time and frequency domains. First, the following time-domain HRV variables were obtained: Mean HRT, SDNN, RMSSD, PSI, and ApEn. SDNN reflects the ability to adjust autonomic balance; lower values indicate ANS dysfunction. SDNN is maintained between 35 and 65 ms; the mean values of girls in their teens such as our patient are approximately 60 ms. RMSSD reflects alterations in autonomic tone mediated by parasympathetic input. PSI reflects the load to the heart based on SDNN or Mean HRT. ApEn has recently been introduced to estimate the complexity of HRV and irregularity within a series of pulses [18–20], with higher values indicating a stressful condition [18, 21]. Second, the following frequency-domain HRV variables were also obtained: TP, VLF, LF, HF, LF norm, HF norm, and LF/HF ratio. TP is the total variance of VLF, LF, and HF [22–24]. VLF, which typically ranges from 0.0033 to 0.04 Hz, is recognized as a supplemental marker of sympathetic function. LF, which ranges from 0.04 to 0.15 Hz, reflects sympathetic activity; HF, which ranges from 0.15 to 0.4 Hz, reflects parasympathetic activity [22, 23, 25]. LF or HF norm is defined as follows: LF or HF norm = 100 × LF or HF/(TP − VLF). LF and HF norm are indices of sympathetic and parasympathetic activity, respectively. LF/HF ratio is an index of autonomic balance and should be maintained between 0.5 and 2.0; higher values reflect sympathetic dominance, while lower ones reflect parasympathetic dominance. Regarding the time domain, the upright position significantly decreased her Mean HRT and PSI and significantly increased her RMSSD. Regarding the frequency domain, her LF norm relatively decreased, and her HF norm increased such that the LF/HF ratio decreased. These findings indicate parasympathetic dominance due to the upright position and are consistent with the fact that OD-associated orthostatic hypotension is caused by sympathetic hypotonia [26]. Regarding the time domain, atropine sulfate administration caused the Mean HRT and PSI to increase significantly in the reclined position while it induced RMSSD and ApEn to decrease significantly. Regarding the frequency domain, the administration induced the LF norm to increase relatively and HF norm to decrease such that the LF/HF ratio increased. These findings indicate relative sympathetic dominance due to atropine-derived parasympathetic blockade and are consistent with the previous report that l-threo-3,4-dihydroxyphenylserine, a precursor of adrenaline, is effective for OD-associated orthostatic hypotension [26].

Local anesthesia induced SDNN and TP to increase significantly and ApEn to decrease significantly in our case. The findings indicate well-regulated ANS function by adrenaline in the local anesthetic. Midazolam administration induced the Mean HRT, ApEn, TP, and LF norm to increase significantly and the HF norm to decrease significantly in our case. These findings indicate sympathetic dominance due to midazolam and are mostly contrary to the previous report that midazolam administration induces significant decreases in Mean HRT and ApEn, TP, LF norm, and HF norm [27]. Furthermore, nitrous oxide inhalation induced the LF norm to decrease significantly and the HF norm to increase significantly such that the LF/HF ratio significantly decreased in our case. These results indicate parasympathetic dominance due to nitrous oxide and are consistent with the previous report that nitrous oxide inhalation inhibits an increase in the LF/HF ratio as a consequence of attenuated LF norm and HF norm constancy [28]. Concomitant use of midazolam and nitrous oxide elicited fully sedative effects for dental management of OD; further detailed investigation is required to elucidate autonomic activity in OD during sedation.

In our case with OD, HRV analysis revealed autonomic dysfunction associated with OD, that is, parasympathetic dominance due to postural change. Our experience emphasizes the importance of HRV analysis with respect to secure and safe dental management of OD patients.

## 4. Conclusion

We experienced a rare case of OD manifested during postural change on the dental chair and intraoperatively monitored by HRV analysis. HRV analysis revealed autonomic dysfunction associated with OD and should become a standard tool for safe and secure dental management of OD.

## Figures and Tables

**Table 1:** Clinical manifestation and scores of frequency-/time-domain HRV variables during dental therapy.

	Therapy onset	Inhale onset	Midazolam administration	Local anesthesia	Teeth extraction	Inhale end	Reclined position	Upright position	Atropine administration and therapy end
Posture	Sitting	Supine	Supine	Supine	Supine	Supine	Supine	Sitting	Supine
Nausea	−	−	−	−	−	−	−	+	−
S/DBP^1^ (mmHg)	106/42	110/55	108/50	131/57	135/69	115/50	108/49	65/25	119/72
Mean HRT^2^ (bpm)	49	47	56	70	49	52	56	51	81
SDNN^3^ (ms)	54.0	67.8	77.7	193.0	73.7	81.4	71.0	86.8	68.2
RMSSD^4^ (ms)	71.2	93.0	84.5	78.5	88.8	84.5	70.3	85.6	16.8
PSI^5^	11.6	13.3	7.0	7.9	7.2	7.2	10.2	7.2	27.1
ApEn^6^	0.866	0.757	0.872	0.273	0.819	0.875	0.946	0.854	0.544
TP^7^ (ms^2^)	1530.0	2948.7	5477.9	33920.5	4196.3	3760.6	2141.8	5215.6	1620.2
VLF^8^ (ms^2^)	757.9	1359.3	4078.6	31187.1	1583.1	1540.3	234.7	1695.0	1138.8
LF^9^ (ms^2^)	300.0	238.8	271.9	1919.1	707.2	1264.9	1416.1	2281.5	426.9
HF^10^ (ms^2^)	472.0	1350.5	1127.4	814.3	1906.0	955.4	491.0	1239.1	54.5
LF norm^11^ (nu)	38.9	15.0	19.4	70.2	27.1	57.0	74.3	64.8	88.7
HF norm^12^ (nu)	61.1	85.0	80.6	29.8	72.9	43.0	25.7	35.2	11.3
LF/HF^13^	0.636	0.177	0.241	2.357	0.371	1.324	2.884	1.841	7.833

HRV: heart rate variability. ^1^Systolic/Diastolic blood pressure. ^2^Heart rate. ^3^Standard deviation of all NN intervals. ^4^Square root of the mean squared differences of successive NN intervals. ^5^Physical stress index. ^6^Approximate entropy. ^7^Total power. ^8^Very low frequency. ^9^Low frequency. ^10^High frequency. ^11^Normalized low frequency. ^12^Normalized high frequency. ^13^Low-frequency/high-frequency ratio.
